# Innovative Approach to Lateral Tibial Plateau Fracture: A Case Study on Anterolateral Submeniscal Arthrotomy With Cement Augmentation and Screw Fixation

**DOI:** 10.7759/cureus.55416

**Published:** 2024-03-02

**Authors:** Siddharth K Patel, Sohael Khan, Ashutosh Lohiya, Saksham Goyal

**Affiliations:** 1 Department of Orthopaedics, Jawaharlal Nehru Medical College, Datta Meghe Institute of Higher Education and Research, Wardha, IND

**Keywords:** anterolateral approach, screw fixation, cement, lateral tibial plateau fracture, submeniscal arthrotomy

## Abstract

Lateral tibial plateau fractures are generally present as depressed fractures. The lateral tibial plateau is more common than the medial tibial plateau, often due to a bumper injury. If depressed fragments are more than 8-10 mm, then surgical management is usually needed. Anterolateral fixation is frequently used for unicondylar lateral tibial plateau fractures. Here, we present an articular depressed lateral tibia plateau fracture in a Schatzker type 3 case. The fracture was managed through an anterolateral approach with sub-meniscal arthrotomy, allowing for direct visualization and subsequent fixation using bone cement and a cannulated cancellous screw. Postoperative imaging confirmed proper reduction, and the patient had a satisfactory outcome..

## Introduction

Tibial plateau fractures comprise an average of 1% of all fracture types. Isolated lateral plateau fractures are the most common pattern [1].

As our understanding of tibial plateau fractures grows, so does the number of available methods, implants, and new techniques. The anatomical restoration of joint alignment and unity with stable fixation is the main objective of fracture reduction on the tibial plateau. Tibial plateau fractures are of the articular type [2]. Due to this, it is challenging to properly reduce and cure articular surface fractures using outdated techniques and implants. Optimal surgical exposure is necessary to accomplish these goals [1].

A single midline anterior exposure used to treat both the medial and lateral condyles has been linked to a significant incidence of wound complications. As a result, a two-incision method for treating bicondylar fractures was developed, utilizing both posteromedial and anterolateral approaches. Consequently, even for solitary lateral plateau fractures, the anterolateral technique has gained popularity [3].

## Case presentation

**Figure 1:**
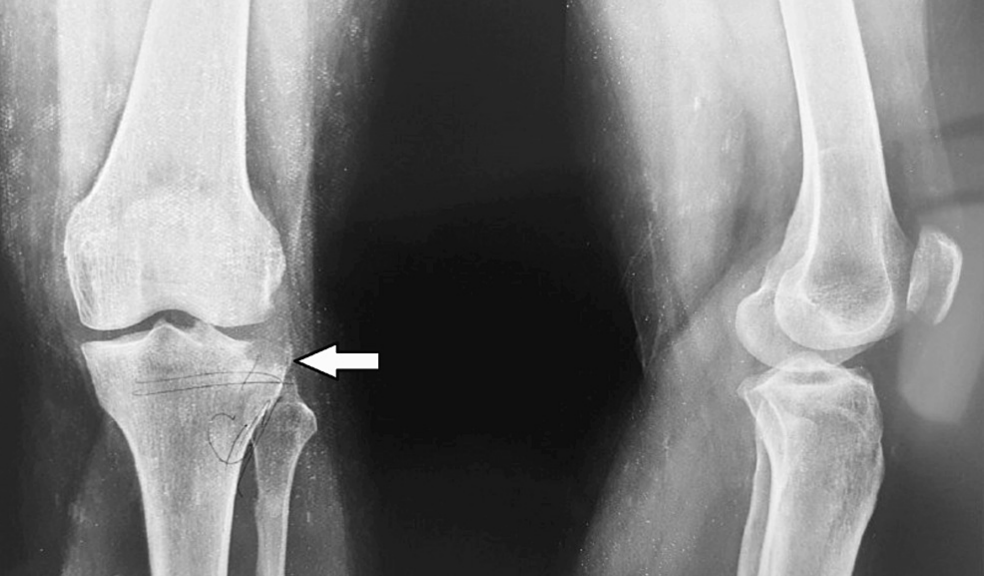
Preoperative X-ray of left knee (anteroposterior and lateral view) showing depressed intraarticular lateral tibial plateau fracture (white arrow)

A 35-year-old female patient presented to the emergency department of our institution with a history of a road traffic accident, sustaining injuries to her left knee. The patient had no other history of hospitalization and no known co-morbidities. An X-ray of the left knee (anteroposterior and lateral views) was done, suggesting a depressed lateral tibial plateau fracture, as shown in Figure 1.

The patient was initially managed with an above-knee slab for stabilization of the fracture. After initial management, the patient was admitted. It was decided to do an anterolateral submeniscal arthrotomy with cement filling and open fixation with cannulated cancellous screws.

**Figure 2:**
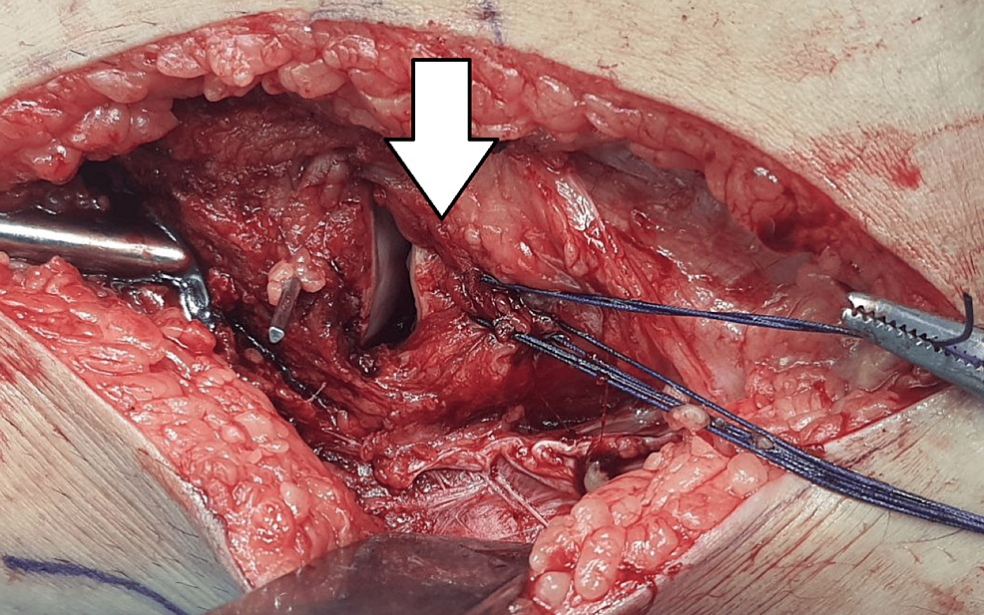
Intra-operative image showing meniscus elevation (white arrow) and intra articular surface of lateral tibia

**Figure 3:**
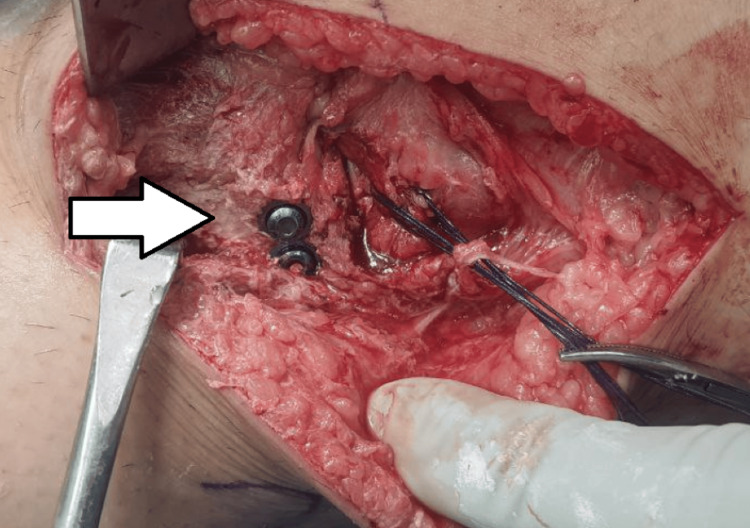
Intra-operative image showing void filled with cement and screw fixation (white arrow)

The patient was taken into the operating theatre room on a table during this procedure. Under spinal anesthesia, the patient was placed in a supine position with a bolster under the left knee. The incision was made straight and lateral to the patella, and then the deep fascia was opened anterior to the iliotibial tract, avoiding injury to the peroneal nerve. The knee joint was exposed by making a horizontal capsulotomy between the deep edge of the meniscus and the tibia. After visualizing the fracture, a submeniscal arthrotomy was performed, and traction sutures using Vicryl 1-0 (Ethicon, Inc., Raritan, New Jersey, United States) were passed at the anterior horn of the lateral meniscus. Traction sutures were pulled proximally, which allowed the articular surface and bone defect to be fully exposed. The bone defect was filled with bone cement and with two cortical screws, fixation of the fracture fragment was done as shown in Figure 2 and Figure 3.

**Figure 4:**
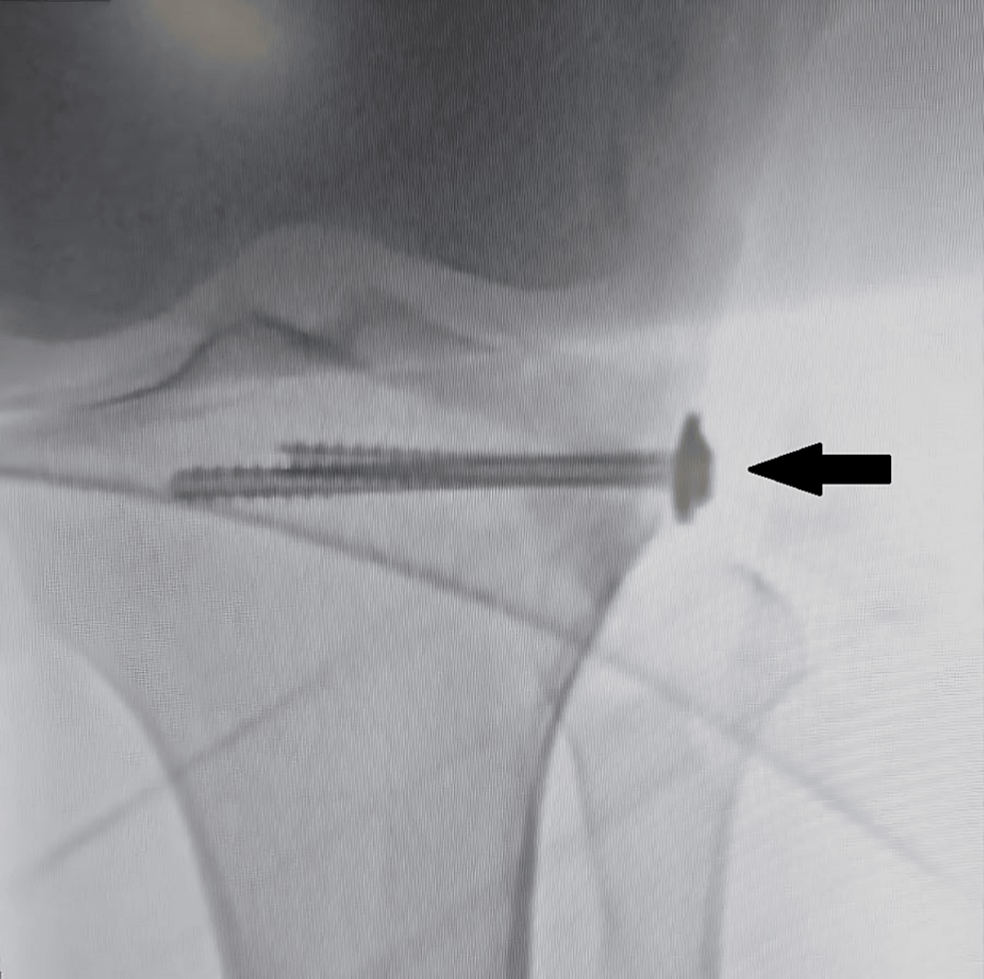
Postoperative X-ray (anteroposterior view) showing void filled with cement and two screws for fixation (black arrow)

The postoperative course was uneventful, and a long knee immobilizer was applied. Postoperative X-rays showed reduction and a maintained articular surface with bone cement and cannulated cancellous screws, as shown in Figure 4.

**Figure 5:**
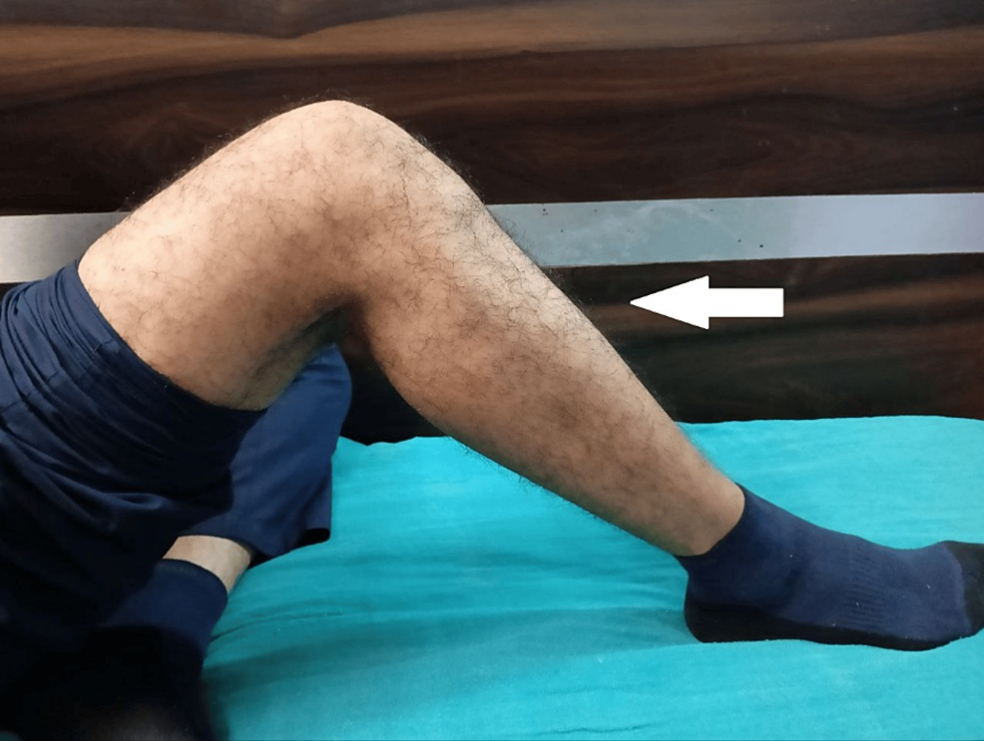
Postoperative six-month follow-up image showing full range of knee movement

**Figure 6:**
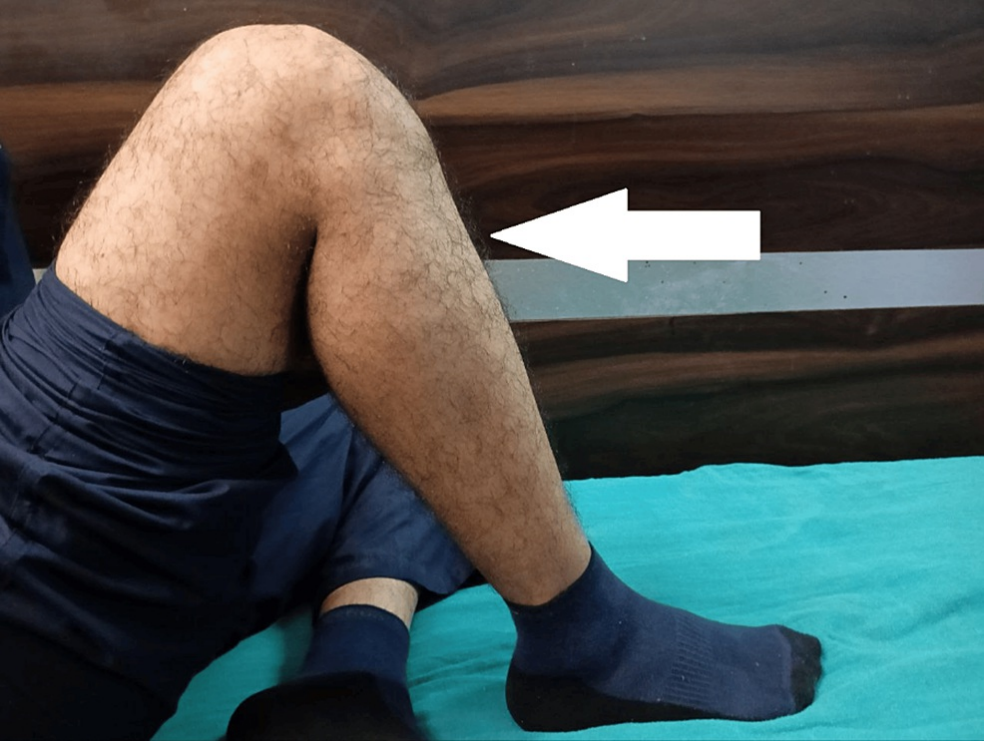
Postoperative six-month follow-up image showing full range of knee movement

Postoperative physiotherapy including quadriceps and hamstring strengthening exercises was started with a straight leg raise, and a long knee immobilizer was applied continuously. The knee range of movement was started after postoperative day 1, and up to 80-90 degree knee flexion was achieved in the first four days. The patient was advised non-weight bearing for four weeks, followed by partial weight bearing as tolerated. Total weight-bearing mobilization was recommended to start after eight weeks. On follow-up after six months, the patient had a full range of movement in the left knee as shown in Figure 5 and Figure 6.

## Discussion

While medial tibial plateau fractures (Schatzker IV-VI) are relatively infrequent, they are considered highly demanding fractures [1,2]. Although prevalent, lateral tibial plateau fractures are the most difficult [3]. Due to the numerous factors that must be considered, including subsidence, displacement, alignment, stability, and soft tissue state, surgical therapy for these fractures can be quite tricky [4-6].

Among them, it has been demonstrated that inadequate reduction and postoperative malalignment are surgeon-dependent prognostic variables that adversely affect functional prognosis and may cause significant morbidity and permanent disability. Consequently, a proper fracture analysis and a preoperative plan are needed. While the significance of articular ridge containment is still being fully understood, it has been noted that insufficient ridge reduction might change the contact forces on the articular surface and cause instability during flexion, which can result in unsatisfactory outcomes [7,8].

In a biomechanical cadaveric investigation, Cuéllar et al. evaluated fracture displacement under torsional, varus, valgus, and axial loads. They found significant fragment movement for all assessed forces and knee flexion angles, except 10 mm pieces under axial load and less than 30° flexion [9].

Compared to the midline method, the anterolateral technique offers a more considerable articular exposure of the lateral tibial plateau. This enhanced exposure might be advantageous when treating fractures that are not amenable to arthroscopic or minimally invasive procedures. It might be most helpful in treating fractures that extend into the posteromedial quadrant of the lateral plateau, fractures that cause the lateral plateau to be extensively comminuted, fractures that involve the tibial spine, or fractures with complicated lateral meniscus tears [1].

Regarding tibial plateau fractures, open reduction and internal fixation combined with a submeniscal arthrotomy yields superior quality reductions, better fracture exposure, and better medium-term outcomes than closed reduction and internal fixation. This could improve this patient group's osteoarthritis symptoms in the long run [10].

The use of rim plates for other columns has been extensively documented in recent years. In this investigation, the fracture was treated with bone cement and cannulated cancellous screws. For the treatment of specific lateral and posterior patterns, available literature suggests using rim plates [11].

## Conclusions

Finally, a potential treatment option for lateral tibial plateau fractures appears to be the combination of submeniscal arthrotomy with cannulated cancellous screws. The method benefits early functional recovery, stability, soft tissue preservation, and fracture reduction accuracy. Nevertheless, more investigation and clinical trials are required to confirm the therapy modality's long-term effectiveness and generalizability.

## References

[1] Gebel PJ, Tryzna M, Beck T, Wilhelm B (2018). Tibial plateau fractures: fracture patterns and computed tomography evaluation of tibial plateau fractures in winter sports. Orthop Rev (Pavia).

[2] Zeltser DW, Leopold SS (2013). Classifications in brief: Schatzker classification of tibial plateau fractures. Clin Orthop Relat Res.

[3] Elsoe R, Larsen P, Nielsen NP, Swenne J, Rasmussen S, Ostgaard SE (2015). Population-based epidemiology of tibial plateau fractures. Orthopedics.

[4] Luo CF, Sun H, Zhang B, Zeng BF (2010). Three-column fixation for complex tibial plateau fractures. J Orthop Trauma.

[5] Kfuri M, Schatzker J, Castiglia MT, Giordano V, Fogagnolo F, Stannard JP (2017). Extended anterolateral approach for complex lateral tibial plateau fractures. J Knee Surg.

[6] Gardner MJ, Yacoubian S, Geller D (2005). The incidence of soft tissue injury in operative tibial plateau fractures: a magnetic resonance imaging analysis of 103 patients. J Orthop Trauma.

[7] Waldrop JI, Macey TI, Trettin JC, Bourgeois WR, Hughston JC (1988). Fractures of the posterolateral tibial plateau. Am J Sports Med.

[8] Brown TD, Anderson DD, Nepola JV, Singerman RJ, Pedersen DR, Brand RA (1988). Contact stress aberrations following imprecise reduction of simple tibial plateau fractures. J Orthop Res.

[9] Cuéllar VG, Martinez D, Immerman I, Oh C, Walker PS, Egol KA (2015). A biomechanical study of posteromedial tibial plateau fracture stability: do they all require fixation?. J Orthop Trauma.

[10] Buckley RE, Schneider P, Duffy PJ, Puloski S, Korley R, Martin CR (2019). A sub-meniscal arthrotomy improves the medium-term patient outcome of tibial plateau fractures. Knee Surg Sports Traumatol Arthrosc.

[11] Zhang BB, Wang BH, Mei J, Luo CF, Zhu Y (2023). Biomechanical study of a new rim plate fixation strategy for two kinds of posterolateral depression patterns of tibial plateau fractures: a finite element analysis. J Orthop Surg Res.

